# Machine Learning Inversion Method for Elastoplastic Constitutive Parameters of Encapsulation Materials

**DOI:** 10.3390/nano16030161

**Published:** 2026-01-25

**Authors:** Mingqi Gao, Tong Hu, Yagang Zhang, Yanming Zhang, Dongyang Lei, You Wang, Yangyang Li, Jian Zhang, Ce Zeng

**Affiliations:** 1 School of Materials and Energy, University of Electronic Science and Technology of China, Chengdu 611731, China; gaomingqi@std.uestc.edu.cn (M.G.); 202421030415@std.uestc.edu.cn (T.H.); liyy@std.uestc.edu.cn (Y.L.); 2 Department of Microwave Integration Center, The 29th Research Institute of China Electronics Technology Group Corporation, Chengdu 610029, China; zhangyanming@cetc.com.cn (Y.Z.); leidongyang@buaa.edu.cn (D.L.); wangyou@stu.xjtu.edu.cn (Y.W.); zhangjian35@cetc.com.cn (J.Z.); zengce@tju.edu.cn (C.Z.); 3 School of Materials Science and Engineering, Tianjin University, Tianjin 300350, China

**Keywords:** electroplated copper, nanoindentation, machine learning, constitutive parameters, inversion

## Abstract

Accurate measurement of material mechanics parameters is crucial for evaluating process quality and product reliability and is a major challenge in the development of 3D heterogeneous integration technology. Aiming to perform high-accuracy measurements of the elastoplastic nonlinear constitutive parameters of microelectronic materials using the nanoindentation testing technique, we take advantage of a neural network to construct a forward characterization model to characterize these response characteristic parameters for different materials, design an improved algorithm for obtaining a reverse iterative solution of the forward characterization model, and develop a material mechanics parameter measurement method to solve overdetermined equations using the least-squares method. This method was further improved by addressing the issues of algorithm stability and solution uniqueness, achieving high-precision and fast reverse solutions for elastoplastic constitutive parameters. The relative error of the material parameters is less than 3% (95% confidence interval), the maximum error is less than 8%, and the inversion convergence error of the key indentation response characteristic parameters is less than 0.1%. The difference between the measured material parameters and the theoretical model in the influence on the process stress of TCV (through ceramic via) products is verified through finite element simulation.

## 1. Introduction

The mechanical properties of materials have a significant impact on the reliability of microelectronic integrated products. Three-dimensional heterogeneous integration (3D HI), a key technology for improving the performance of microelectronic products, needs to address the complex issue of compatibility between different malarial systems. The characteristics of microelectronic materials are closely coupled with process methods; therefore, accurate measurement of material mechanical parameters is crucial for evaluating process quality and product reliability and is a major challenge in the development of 3D HI technology. Traditional material mechanics testing methods require the preparation of macroscopic (cm level) test samples that meet the requirements of mechanical testing machines. However, this method is not feasible for the microelectronic materials formed during manufacturing processes, such as μm scale wiring and interconnection hole materials (usually gold or copper) formed during the electroplating process, in situ grown dielectric films [1], etc. The nanoindentation testing technique utilizes high-resolution sensing devices to continuously monitor payload (*P*) and displacement (*h*) during the indentation process and solves the mechanics parameters of the material based on the acquired *P*–*h* curve [2]. As nanoindentation testing only requires μm scale samples and supports near non-destructive in situ testing, it has gradually become a fast and simple mechanical performance testing method in the field of microelectronic packaging in recent years [3,4], widely used in the research of TSV (through silicon via) [5,6,7], TGV (through glass via) [8], MEMSs (micro-electro-mechanical systems) [9,10], nano metal bonding [11], etc.

Through the adoption of the widely accepted Oliver Pharr model [12] and the Cheng–Cheng method [13], nanoindentation testing can now be used to reliably acquire the elastic modulus and hardness values of materials [14]. To accurately design a method for determining the reliability of microelectronic products, it is necessary to obtain the key elastoplastic nonlinear constitutive parameters of materials, such as yield strength (*σ_y_*) and hardening index (*n*). However, there is a complex high-order nonlinear coupling relationship between these mechanical parameters and nanoindentation test data [15], which is difficult to calculate directly using analytical methods. Early research mainly relied on dimensional analysis [16,17,18], in which functions used to fit material parameters are constructed based on a certain amount of testing/simulation data. Since these methods are limited by data sources and the complexity of dimensionless functions, achieving high calculation accuracy is difficult, and the calculation accuracy varies significantly in different material parameter ranges. Another approach is to combine dimensional analysis with finite element method (FEM) for iterative calculation. This approach can improve calculation accuracy [11,19] but has the drawbacks of high computational cost and long calculation time, making it unsuitable for practical applications. In the bid to expand the application scope of the nanoindentation testing technique, explorations into high-precision, high-confidence, and fast nonlinear material parameter inversion methods are urgently needed.

Deep learning is a data-driven method for extracting patterns and features and has achieved great success in solving complex nonlinear problems such as image processing and natural language processing. Deep learning has unparalleled advantages in complex nonlinear modeling compared with traditional methods and is expected to provide a better route for the inversion of material nonlinear constitutive parameters in nanoindentation testing [20]. Marimuthu et al. [21] conducted regression modeling on *P*–*h* curve sampling data and material parameters and introduced a physical constraint equation of unloading initial slope (*S*) to construct an inversion method based on a physics-informed artificial neural network (PI-ANN). Park et al. [22] determined the minimum input feature parameters *(h_max_*, *S*, *W_p_*/*W_t_*, and *C*) and training data scale through correlation analysis. The application of transfer learning has also greatly reduced the scale of high-precision training dataset [23,24]. Jiao et al. [25] systematically studied the solution uniqueness problem in the reverse solution of elastoplastic constitutive parameters from the perspective of machine learning and proposed the use of pile-ups generated during the indentation process as the input feature of machine learning. Previous research has fully demonstrated the potential of deep learning in the inversion of nanoindentation data, but the high sensitivity of nanoindentation curves to material parameters and the solution uniqueness of inversion [25,26] make obtaining accurate and stable solutions using deep learning difficult, which thus necessitates in-depth research.

This study proposes a novel method for measuring material mechanics parameters based on the principle of solving overdetermined equations using the least-squares method. First, a forward characterization model of the response characteristic parameters of the nanoindentation process under different material mechanics parameters is constructed based on a neural network. Then, the least-squares inverse iteration is performed based on the forward characterization model to obtain the solution. Thus, the problem of measuring material parameters is transformed into the problem of finding the least-squares optimal solution of nonlinear overdetermined equations. This method is further improved by addressing the issues of algorithm stability and solution uniqueness. The method proposed in this paper avoids a major problem in the reverse solution of material parameters: the solution being difficult to obtain because the analytical relationship between material parameters and indentation experimental data is hard to establish. This paves the way for achieving rapid, accurate, and stable extraction of material mechanical parameters from nanoindentation experimental data. FEA simulation data demonstrates that the proposed algorithm can yield reverse solution results with high accuracy (both the strengthening index *n* and yield strength *σ_y_* are <5%) and high stability and can avoid the multi-solution problem (multiple calculations yielding the same solution, and a convergence mean square error (MSE) < 1 × 10^−8^), which is superior to the results reported in References [11,19,23].

## 2. Methodology

### 2.1. Constitutive Model

**Figure 1:**
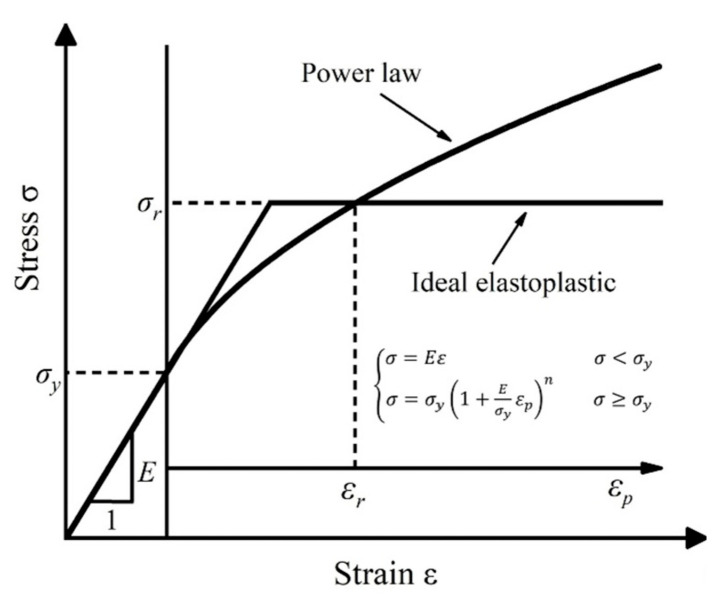
Power law stress–strain curve of the materials beyond yield strength.

This study focuses on a typical elastoplastic constitutive model featuring linear elasticity governed by Hooke’s Law and power law hardening behavior. This model corresponds to the elastoplastic constitutive equation presented in Equation (1), with its associated stress–strain curve illustrated in Figure 1. Given that most materials—particularly metals—adhere well to this model, the research is dedicated to inverting the power law constitutive parameters of materials using nanoindentation test data [27].

(1) σ={          E·ε                                   σ≤σy R·εn=σy(1+Eσyεp)n          σ≤σy

where *E* is Young’s modulus (Pa);

*R* is the strength (Pa);
*σ_y_* is the yield strength (Pa);
*ε* is the total strain;
ε_p_ is the plastic strain;
*n* is the strain strengthening index.

### 2.2. Reverse Analysis Framework

**Figure 2:**
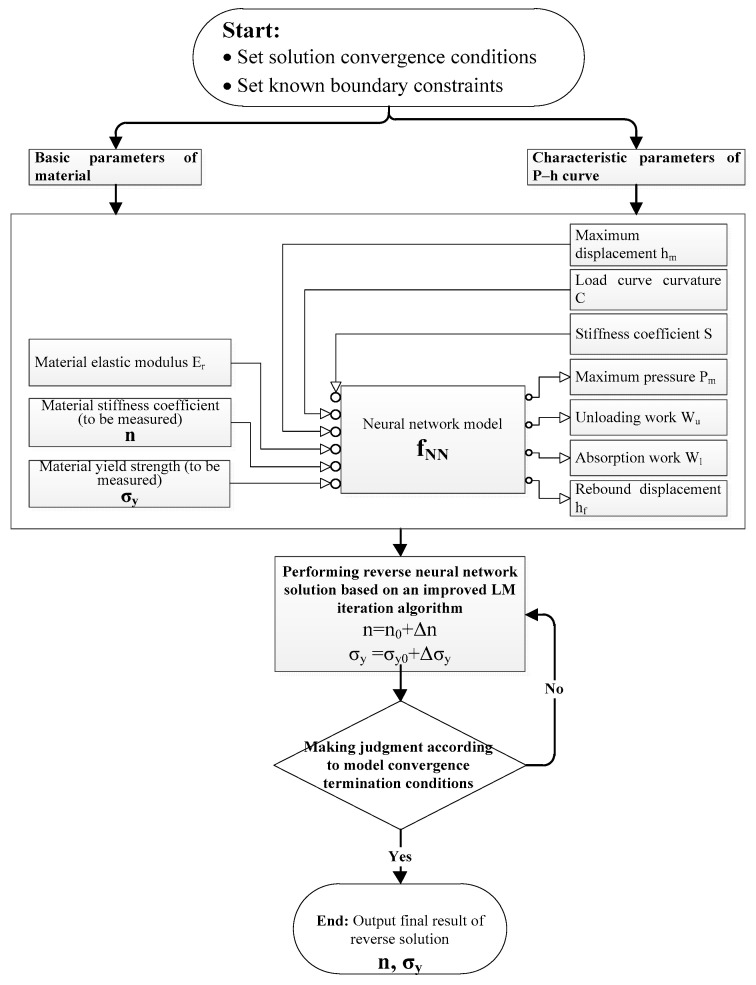
The overall framework for reverse calculation of the material mechanics parameter based on the principle of least-squares solution for overdetermined equations.

The overall framework of the material mechanics parameter reverse calculation method based on the principle of solving overdetermined equations using the least-squares method proposed in this article is shown in Figure 2. It mainly includes a neural network model constructed based on the basic material parameters and the characteristic parameters of the pressure–displacement curve, and a neural network reverse solution algorithm based on the improved LM (Levenberg–Marquardt) iteration algorithm.

**Figure 3:**
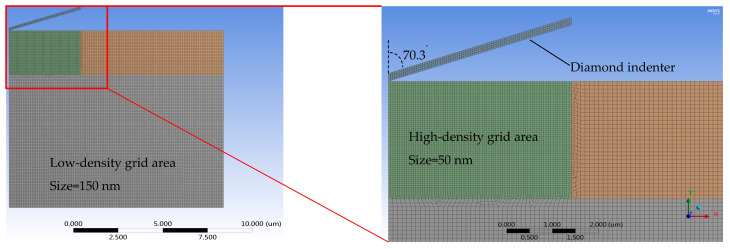
The finite element simulation diagram of neural network model construction.

In this study, finite element simulation was used to conduct a virtual experiment of nanoindentation, obtaining a large amount of high-precision data with a low cost for the construction of the neural network model. The simulation tool used in the virtual experiment was the static mechanical analysis module of the commercial software Ansys Workbench. The tetrahedral Berkovich indenter was substituted with a 2D axisymmetric model with the same projected area to accelerate the simulation speed [28]. The grid density in the area near the indenter and the pressed material was set to 50 nm, and the number of cells was 18,396. The mesh size in the farther regions was set to 150 nm (Figure 3). The mesh quality of the finite element simulations performed via Ansys in this study has undergone mesh independence analysis. This adequately ensures that the extracted simulation result data (e.g., *S*, *C*, and *P_m_*) are not affected by the model’s mesh division, with the actual discrepancy less than 0.1%. The minimum number of calculation steps during the loading and unloading stages was set to 1000. Considering that Poisson’s ratio (*ν*) has a small impact on the calculation results, it was set to a fixed value of 0.32. Five variables were set in the virtual experiment, and the range of values and factor levels were determined based on the possible elastoplastic material parameters of electroplated copper (Table 1). A total of 2016 rounds of a full-factor orthogonal DOE simulation experiment were conducted, each round yielding one piece of *P*–*h* data. Automatic creation of the simulation model and submission of the simulation results were achieved by writing Python (The version is Iron Python 2.7.11) scripts.

**Figure 4:**
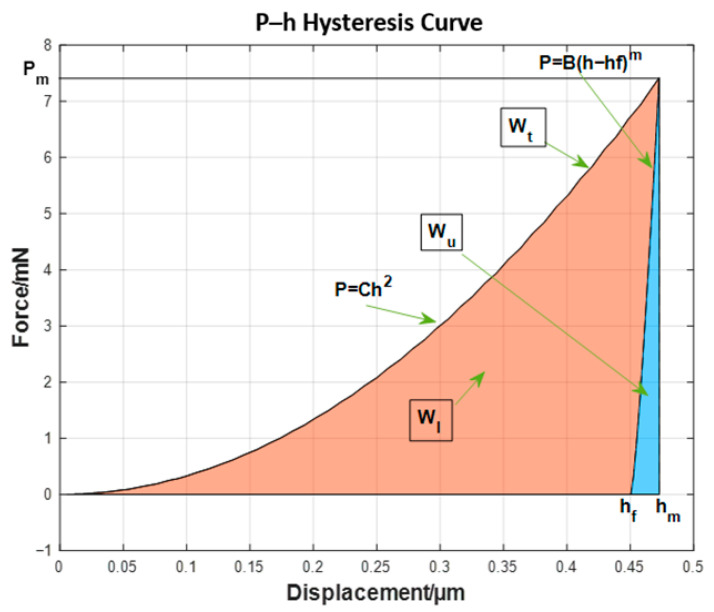
Typical nanoindentation *P*–*h* curve of each group of materials.

The indentation *P*–*h* curve data corresponding to each group of material parameters was obtained through a virtual simulation experiment (Figure 4). The curve feature parameters were defined and extracted to train the neural network model, including the following:

(a) *P_m_* is the maximum pressure, mN.
(b) *h_m_* is the maximum displacement, μm.
(c) *h_f_* is the residual displacement, μm.
(d) *C* is the loading curvature, calculated using the equation $C=\frac{P_{m}}{h_{m}^{2}}$.
(e) *S* is the stiffness coefficient, defined and calculated using Equation (2) based on the unloading curve, where parameters *B* and *m* are obtained through fitting according to the unloading power function. The stiffness coefficient can also be obtained directly using a nanoindentation instrument; thus, *m* and *B* can be calculated directly using Equations (3) and (4):
  (2) $$S={\left.\frac{dP}{dh}\right|}_{h=h_{m}}=Bm{\left(h_{m}-h_{f}\right)}^{m-1}$$
  (3) $$m=\frac{S\left(h_{m}-h_{f}\right)}{F}$$
  (4) $$B=\frac{F}{{\left(h_{m}-h_{f}\right)}^{m}}$$
(f) *W_t_* is the total indentation work, which is the area under the *P*–*h* curve when the indentation reaches its maximum displacement. It can be calculated using Equation (5):
  (5) $$W_{t}=\int_{0}^{h_{m}}Pdh=\int_{0}^{h_{m}}Ch^{2}dh=\frac{1}{3}Ch_{m}^{3}$$
(g) *W_u_* is the unloading work, which is the area under the unloading curve. It can be calculated using Equation (6):
  (6) $$W_{u}=\int_{h_{f}}^{h_{m}}Pdh=\int_{h_{f}}^{h_{m}}B{\left(h-h_{f}\right)}^{m}dh=\frac{1}{m+1}B{\left(h_{m}-h_{f}\right)}^{m}$$
(h) *W_l_* is the absorption work, $W_{l}=W_{t}-W_{u}$.

### 2.3. Neural Network Structure and Training Algorithm

**Figure 5:**
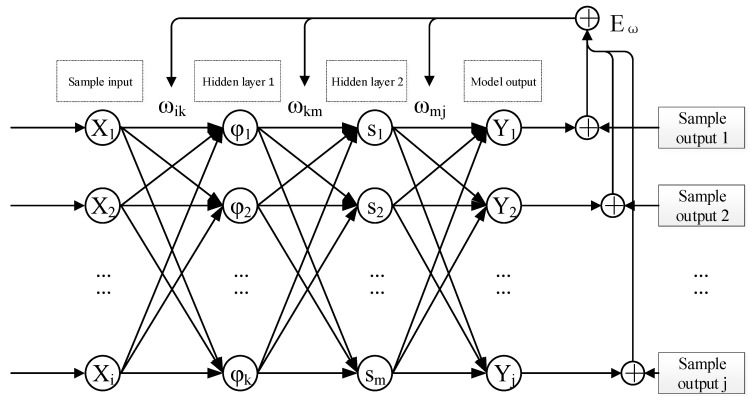
Structure of the fully connected neural network and training error propagation path.

(1) The neural network used in this study is a fully connected BP neural network, which has the structure shown in Figure 5. It has an input dimension of 6 and an output dimension of 4. The network consists of two hidden layers: the first hidden layer is configured with 30 neurons and adopts the hyperbolic tangent (tanh) function as its activation function; the second hidden layer contains 20 neurons with the sigmoid function as its activation function. The output layer employs a linear activation function.

The first hidden layer of the neural network uses the tanh function (7), and the second hidden layer of the neural network uses the sigmoid function (8):

(7) $$\varphi \left(\cdot \right)=\frac{2}{\left(1+e^{-2\alpha }\right)}-1$$

(8) $$s\left(\cdot \right)=\frac{1}{\left(1+e^{-\alpha }\right)}$$

where $\alpha^{\left(l\right)}=\sum_{i=1}^{h_{l-1}}\left(z_{i}^{\left(l-1\right)}\omega_{i}^{\left(l-1\right)}\right)+b^{\left(l\right)}$ is the weighted sum (with added bias) of all outputs of the previous layer that is input into the neuron function of the *l*-th layer.

(2) Training algorithm with Bayesian regularization

To improve the generalization prediction ability of the model constructed based on the neural network proposed in this paper and to prevent overfitting, we adopted Bayesian regularization to train the algorithm. In addition, the L2 regularization term is constructed by adding the sum of squares of the model parameters based on the MSE loss function to limit the size of the model parameters, thereby limiting the number or magnitude of the model parameters and improving the generalization performance of the model [29,30]. Thus, the loss function *E_ω_* used to train the neural network is as follows:

(9) $$\begin{array}{cc} E_{\omega } & =\frac{\lambda }{2N}\sum_{n=1}^{N}\sum_{m=1}^{M}{\left(f\left(x\right)\right)}^{2}+\frac{\left(1-\lambda \right)}{2N}\sum_{n=1}^{N}f{\left(\omega \right)}^{2}\\ & =\frac{\lambda }{2N}\sum_{n=1}^{N}\sum_{m=1}^{M}{\left(y_{nm}-t_{nm}\right)}^{2}+\frac{\left(1-\lambda \right)}{2N}\sum_{n=1}^{N}{\left\|\omega \right\|}^{2} \end{array}$$

where *λ* is the regularization hyperparameter, which is set to 0.5 in this study.

For data partitioning, the training, validation, and test sets accounted for 70%, 15%, and 15% of the total dataset, respectively. During training, input features were linearly normalized to the range of [−1, 1] (the specific range was adjusted based on the sensitivity differences in each parameter; see Table 2 for details), while output targets were normalized to [0.1, 0.9]. The model adopted mean squared error (MSE) as the performance evaluation metric, with the optimization objective of minimizing the error. The training algorithm used was Bayesian regularization (trainbr), with the relevant hyperparameter settings as follows: maximum number of training epochs = 1000, minimum gradient threshold = 1 × 10^−7^, initial damping coefficient (Mu) = 0.005, damping coefficient decay ratio = 0.1, growth ratio = 10, and maximum damping coefficient = 1 × 10^10^.

### 2.4. Reverse Solution Algorithm

(1) Definition of the reverse solution model

The above neural network (as shown in Figure 5) can be written as a function mapping the representation of independent vs. dependent variables: $f_{ANN}\left(\vec{X}\right)=\vec{Y}$, where

(10) $$\vec{X}=\left[E_{r},n,\sigma_{y},h_{m},C,S\right]$$

(11) $$\vec{Y}=\left[P_{m},W_{u},W_{l},h_{f}\right]$$

The equation is further divided into a system consisting of four equations. This system of nonlinear equations mapping the input and output variables is the aforementioned neural network model constructed in this study.

(12) $$\left\{\begin{array}{c} f_{ANN}\left(E_{r},n,\sigma_{y},h_{m},C,S\right)=P_{m}\\ f_{ANN}\left(E_{r},n,\sigma_{y},h_{m},C,S\right)=W_{u}\\ f_{ANN}\left(E_{r},n,\sigma_{y},h_{m},C,S\right)=W_{l}\\ f_{ANN}\left(E_{r},n,\sigma_{y},h_{m},C,S\right)=h_{f} \end{array}\right.$$

In the indentation experiment, *n* and *σ_y_* are unknown variables to be measured, serving as variables to be solved in the system of equations. According to the relationship between the number of equations and the number of unknown variables, when the number of equations is greater than the number of unknowns, the system of equations is overdetermined and has no exact solution. Therefore, when other input and output variables are known, the problem is transformed into finding the least squares solution of the above neural network equation system.

This study proposes an improved iterative optimization algorithm designed based on the LM algorithm for a reverse solution of the nonlinear equation system composed of the aforementioned neural network. The optimization objective function is as follows:

(13) $$E_{r}=\frac{1}{N}\sum_{n=1}^{N}\sum_{j=1}^{K}{\left(f_{j}\right)}^{2}=\frac{1}{N}\sum_{n=1}^{N}\sum_{j=1}^{K}{\left(t_{nj}-y_{nj}\right)}^{2}$$

where *y* is the predicted output of the model; *t* is the output of sample data; *K* is the number of output dimensions, i.e., the number of equations; and *N* is the number of sample data groups. It is feasible to obtain the solution of the equation system by combining *N* groups of data. However, in normal experiments, there is no guarantee that the material parameters corresponding to each group of test data are the same. If the data groups are combined, there will be a problem of imbalanced solution conditions and failure of convergence. Therefore, *N* is selected as 1, and *N* greater than 1 is only used as a reference candidate. Combining the data groups is feasible when the material parameters of each set of test data are the same.

Therefore, the *n* and *σ_y_* values (obtained after reverse iteration) that yield the smallest value from the optimization objective function are the material parameters to be obtained through inversion based on the nanoindentation *P*–*h* curve.

(2) Improvement of the inversion objective function

To solve the system of equations constructed based on a neural network, we designed an improved MSE objective function, shown in Equation (14), in which the weight factors can be adjusted in multi-objective optimization:

(14) $$E_{r}=\frac{\left[\begin{array}{cccc} \lambda_{1} & \lambda_{2} & \lambda_{3} & \lambda_{4} \end{array}\right]}{\sum_{i=1}^{4}\lambda_{i}}\times \left[\begin{array}{c} \frac{1}{N}\sum_{n=1}^{N}{\left(Pm_{n}-t_{n}^{\left[Pm\right]}\right)}^{2}\\ \frac{1}{N}\sum_{n=1}^{N}{\left(Wu_{n}-t_{n}^{\left[Wu\right]}\right)}^{2}\\ \frac{1}{N}\sum_{n=1}^{N}{\left(Wl_{n}-t_{n}^{\left[Wl\right]}\right)}^{2}\\ \frac{1}{N}\sum_{n=1}^{N}{\left(hf_{n}-t_{n}^{\left[hf\right]}\right)}^{2} \end{array}\right]$$

where *λ* is the error weight coefficient in the range from 0 to 1 (but cannot be 0 at the same time), used to control the contribution of each output error to the iterative solution.

(3) Improvement of the inversion iteration algorithm

For the neural network with an input of ***X***, the iterative solution is based on the following formula:

(15) $$X_{new}=X_{pre}+h_{lm}$$

where ***h****_lm_* is the iteration step size of each input variable. According to the LM algorithm, the calculation formula for the iteration step size is as follows [31,32]:

(16) $$h_{lm}=-{\left(H+\mu I\right)}^{-1}\times g$$

where *μ* is the damping parameter in the LM algorithm; *H* represents the Hessian matrix $H=J^{T}\times J$; and ***g*** represents the gradient vector of the loss function $g=J^{T}\times f$, where *f* is the error between the model output and the sample output defined in the loss function.

To achieve a flexible definition of the variables to be solved through inversion in input ***X***, we improved the LM algorithm in the following way: We dot multiplied a logical mask row vector ***M*** on the basis of the original Jacobian matrix, where each element of ***M*** has a value of 0 or 1, and the length is equal to the dimension number of input parameter ***X***. By assigning a value of 1 to an element ***M***, iterative updating of the input parameter at the corresponding element position is controlled; if the value of 0 is assigned, the iteration step size is always equal to 0, which is equivalent to keeping the initial value of the corresponding element position constant. Thus, the reverse iterative solution of the remaining unknown variables can be achieved when some input quantities and all output quantities are known.

(17) $$J=M\cdot \left[\begin{array}{c} \begin{array}{cccc} \frac{\partial f_{1}}{\partial X_{1}}, & \frac{\partial f_{1}}{\partial X_{2}}, & \ldots , & \frac{\partial f_{1}}{\partial X_{n}} \end{array}\\ \vdots \\ \begin{array}{cccc} \frac{\partial f_{j}}{\partial X_{1}}, & \frac{\partial f_{j}}{\partial X_{2}}, & \ldots , & \frac{\partial f_{j}}{\partial X_{n}} \end{array} \end{array}\right]$$

The convergence criterion for the training process was defined such that the iteration terminates if any of the following conditions is satisfied: reaching the maximum number of training epochs, the loss function falling to the preset target, the gradient norm dropping below the minimum threshold, or the damping coefficient exceeding the upper limit. This setup is designed to balance training efficiency and the stability of model convergence.

## 3. Application Verification and Discussion

### 3.1. Model Evaluation

**Figure 6:**
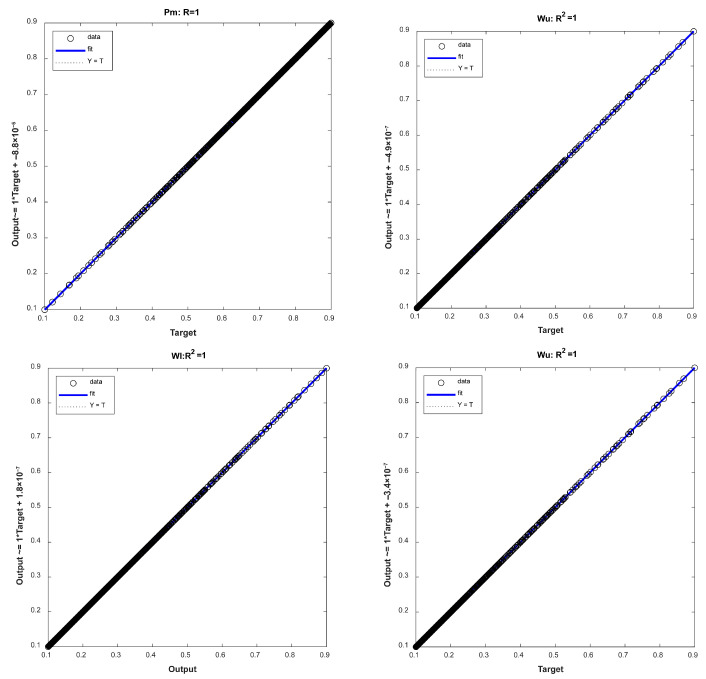
R^2^ evaluation of the output in various dimensions for the training results of neural network models used for a reverse solution.

The coefficient of determination R^2^ was used to evaluate the training results of the neural network model used for a reverse solution, which is defined by Equation (18). According to common practice in neural network training, the input and output variables were normalized to the [−1, 1] interval before starting model training. The training ended when the convergence accuracy MSE reached around 1 × 10^−9^. Figure 6 shows that the R^2^ of every output variable of the model relative to the sample data reached 1, indicating that the model can characterize the correlation between the selected inputs and outputs very well.

(18) $$R^{2}=1-\frac{\sum_{i=1}^{N}{\left(y_{i}-y_{i}^{*}\right)}^{2}}{\sum_{i=1}^{N}{\left(y_{i}-{\bar{y}}_{i}\right)}^{2}}$$

When the proposed model is used for reverse solution testing on the to-be-solved input variables *n* and *σ_y_*, problems of unstable convergence at some points and multiple solutions can be found. Due to the phenomenon of non-convex optimization in multi-objective and multivariate nonlinear iteration, complex problems such as difficult convergence and multiple extrema are common, which can affect the stability and uniqueness of the reverse solution of model.

The problem of solution stability is manifested in the following phenomenon: significant difference in convergence accuracy exists between the results of different iterations of the to-be-solved variables if different initialization values are used. As a result, failure to converge or premature termination of the iteration can occur, making it impossible to stably obtain the inversion convergence solution every time.

The problem of solution uniqueness is manifested in the following phenomenon: the solution always converges to relatively high precision for the models obtained from different training batches or when the same model is assigned different initial conditions, but each time, the solution is significantly different from the previous one.

**Figure 7:**
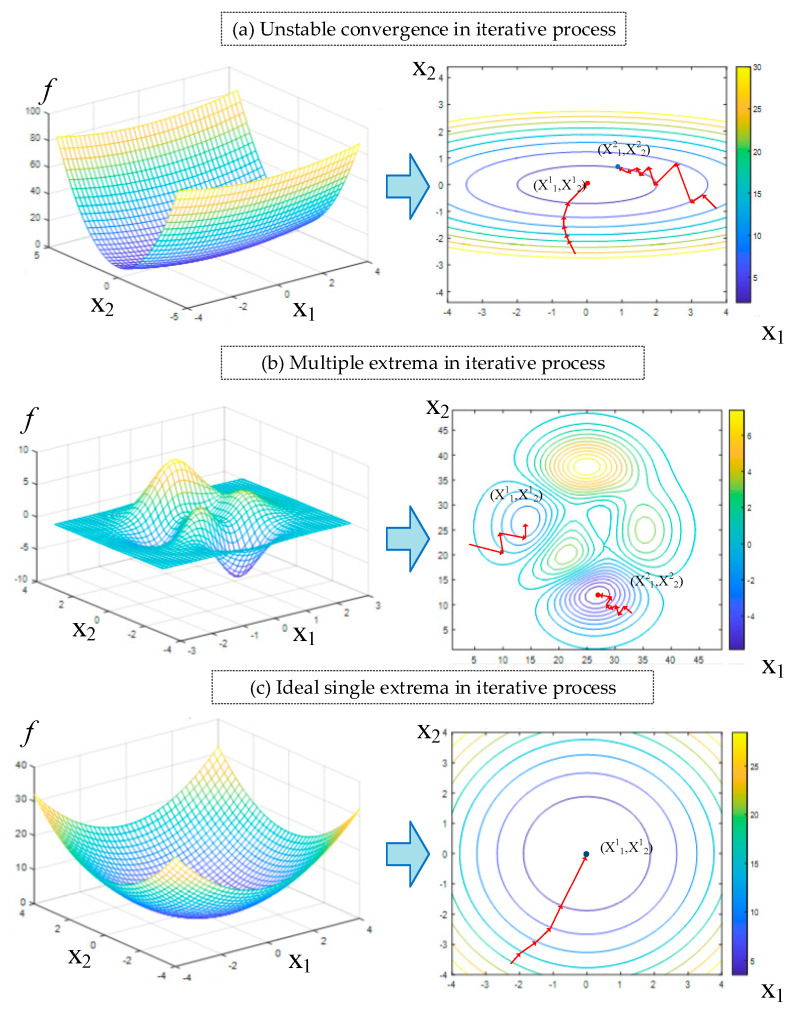
The solution instability and non-uniqueness during the reverse solution process for the parameters *n* and *σ^y^* using the R^2^ evaluation neural network model.

Taking the iterative optimization process of the binary variable as an example, the variation in unstable iterative convergence gradients is shown in Figure 7a, the iterative convergence process of non-unique solutions is shown in Figure 7b, and the ideal case is shown in Figure 7c. The solution can always converge to the same extremum point regardless of the initialization values, and the solution is stable and unique.

Based on the above analysis, we devised measures to address the problems of unstable and non-unique solutions in the reverse solution process: evaluate and analyze the sensitivity of input and output parameters using global sensitivity [33], and on this basis, adjust the model training process.

Global sensitivity, represented by *Sen_i_*, is defined as the degree to which an output parameter is affected by a certain one-dimensional input ***X****_i_* under the variation in disturbances to other inputs *x*_*j*≠*i*_ in the entire sample analysis space. For each pair of input–output samples ($\vec{X_{k}}$, $\vec{Y_{k}}$), the derivative at that sample point is calculated and then summed up. The direct summation of derivatives at each point reflects the directional polarity of the influence, which is defined as vector sensitivity; the accumulation of the absolute value of the derivative at each point reflects the total degree of influence, which is defined as scalar sensitivity. The vector sensitivity and scalar sensitivity can be calculation using the following equation:

(19) $$Sen_{i}=\left\{\begin{array}{c} \begin{array}{cc} \sum_{k=1}^{D_{S}}\left|\frac{\partial {\vec{Y}}_{k}}{\partial X_{ik}}\right| & ,scalar \end{array}\\ \begin{array}{cc} \sum_{k=1}^{D_{S}}\frac{\partial {\vec{Y}}_{k}}{\partial X_{ik}} & ,vector \end{array} \end{array}\right.$$

When the input parameters are normalized to the same range, sensitivity analysis reveals that different input parameters have significantly different influences on the outputs regarding sensitivity. The parameters with lower sensitivity (mainly *E_r_*, *n*, *σ_y_*, and *S*) have a weak relationship with the model output. This indicates that when the normalization range is uniform, there is still a significant scale difference in the influence of the parameters on the outputs. During the model reverse solution process, the influence of initial values can easily cause the model to become trapped in local optima, resulting in convergence failure and unstable solution. Based on the relative relationship of sensitivity under the uniform normalization range for all parameters, we can adjust the hyperparameters by setting narrow normalization range for parameters with lower sensitivity, as shown in the table below.

**Figure 8:**
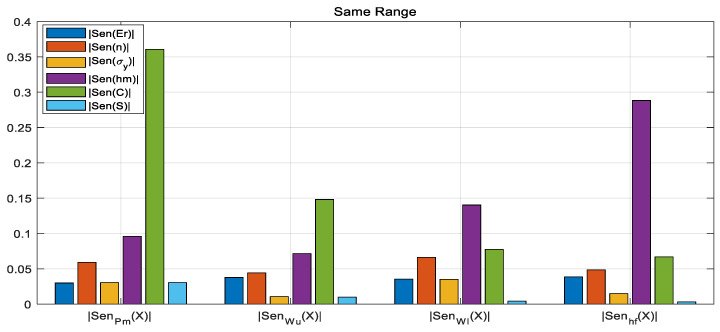
The globally normalized scalar sensitivity for solution instability and non-uniqueness in the R^2^ evaluation exhibits an overall imbalance.

**Figure 9:**
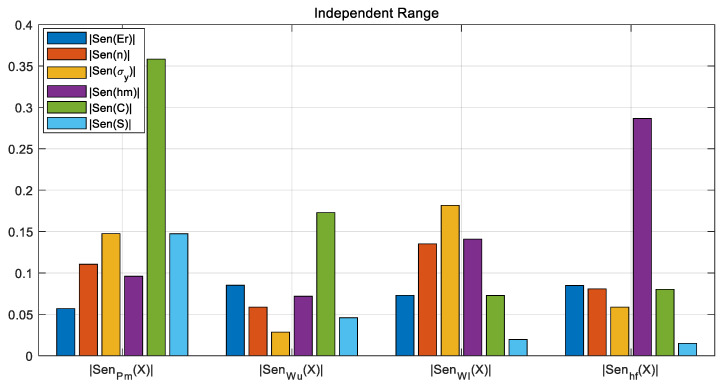
The globally normalized scalar sensitivity for solution instability and non-uniqueness in the R^2^ evaluation exhibits a balanced overall distribution after adjusting compensation for enhanced parameter sensitivity.

By comparing Figure 8 and Figure 9, we can see that parameter sensitivity can be enhanced through adjustment and compensation, achieving better overall balance.

A comparison of the sensitivity values before and after adjusting the normalization ranges is presented in Table 3. The effectiveness of the compensation technique proposed in this paper can be verified from the table as follows:

The sensitivity of key constitutive parameters is significantly enhanced: In the balanced sensitivity mode, the influence of material constitutive parameters (yield strength *σ_y_* and hardening exponent *n*) on most output variables is substantially strengthened. For instance:

(a) The sensitivity of σy to the maximum load Pm rose from 0.0232 to 0.1475 (an increase of approximately 535%).

(b) The sensitivity of the hardening exponent *n* to *P_m_* also increased from 0.0495 to 0.1105. This demonstrates that the proposed technique effectively improves the inversion algorithm’s capability to identify the core parameters to be solved.

The sensitivity distribution is more balanced, alleviating the parameter coupling issue: In the unbalanced mode, some parameters exhibit extremely low sensitivity (e.g., the sensitivity of stiffness *S* to residual depth *h_f_* is merely 0.0035), which tends to make the inversion process susceptible to measurement noise or prone to converging to local solutions. After compensation, the sensitivity distribution of all parameters becomes more uniform (e.g., the sensitivity of *S* to *h_f_* is increased to 0.0149), which helps enhance the numerical stability of the inversion system and the reliability of its solutions.

The compensation technique optimizes the exploration efficiency of the parameter space: Balanced sensitivity ensures that the weight of all input parameters’ influence on the outputs is of the same order of magnitude, preventing certain parameters from being “ignored” by the algorithm due to dimensional or numerical range discrepancies. Thus, the inversion process is guaranteed to conduct more efficient and comprehensive searches across the entire parameter space.

In addition, a sensitivity analysis shows that all four selected output variables are correlated with the input variables, and the higher the sensitivity, the stronger the correlation. However, the variables *n* and *σ_y_* have relatively low sensitivities compared with other input parameters. When the outputs are partially used (the number of equations in equation system (12) >2 but <4), it is impossible to iteratively converge to an exact solution, and there is a problem of multiple solutions. Therefore, all four output variables are used for solving unknown variables.

**Figure 10:**
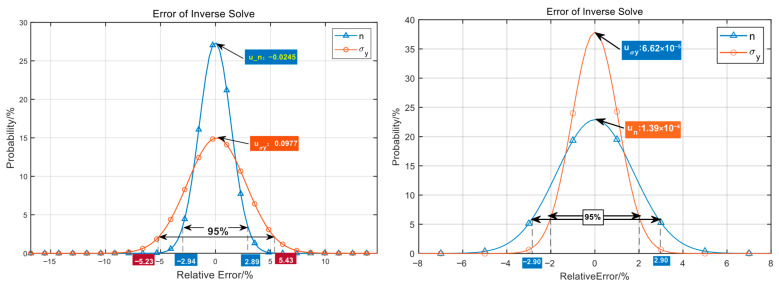
Evaluation of relative error of parameter reverse solution (the left figure uses the uniform normalization range, and the right figure uses normalization of sensitivity-based compensation).

Through the above two adjustment steps, stability and uniqueness of the solution are achieved. As can be seen from the comparison of the relative error distribution for the reverse solution shown in Figure 10, the 95% confidence percentage error is improved from 5% to 3%, and the maximum error is controlled within 8%.

### 3.2. Physical Rationality and Accuracy Verification of Solution

To verify that the numerical iterative solution conforms to physical constraints, a system of equations is constructed for the elastoplastic constitutive curve’s intersection point under the conditions of *n* = 0 and *n* ≠ 0 (Figure 1), and the characteristic strain calculation formula at the intersection point of the curves is obtained (20). The solutions that conform to physical constraints should satisfy the intersection point formula:

(20) $$\varepsilon_{r}=\frac{\sigma_{y1}}{E_{r}}\left[{\left(\frac{\sigma_{y2}}{\sigma_{y1}}\right)}^{{1/n}_{1}}-1\right]$$

where the characteristic stress σ_r_ at the intersection point is *σ_y_*_2_ when *n*_2_ = 0.

Based on the prior physical constraints of the intersection point of the stress–strain curve, the characteristic parameters *E_r_*, *P_m_*, *h_m_*, *W_l_*, and *C* can be extracted during the loading stage of the material *P*–*h* test curve under the condition of *n* ≠ 0. Then, the characteristic stress *σ^R^_r_* at *n* = 0 (i.e., ideal plastic material) can be solved through model inversion. Furthermore, based on the engineering experience value of *ε_r_* = 0.033 (0.025–0.033) and the material parameters n and σ_y_ obtained through inversion, the characteristic stress *σ^R^_r_* of the intersection point can be obtained using the intersection point formula. When the characteristic stress values calculated using the two methods are close (difference < 10%), the solution conforms to the physical constraints.

**Figure 11:**
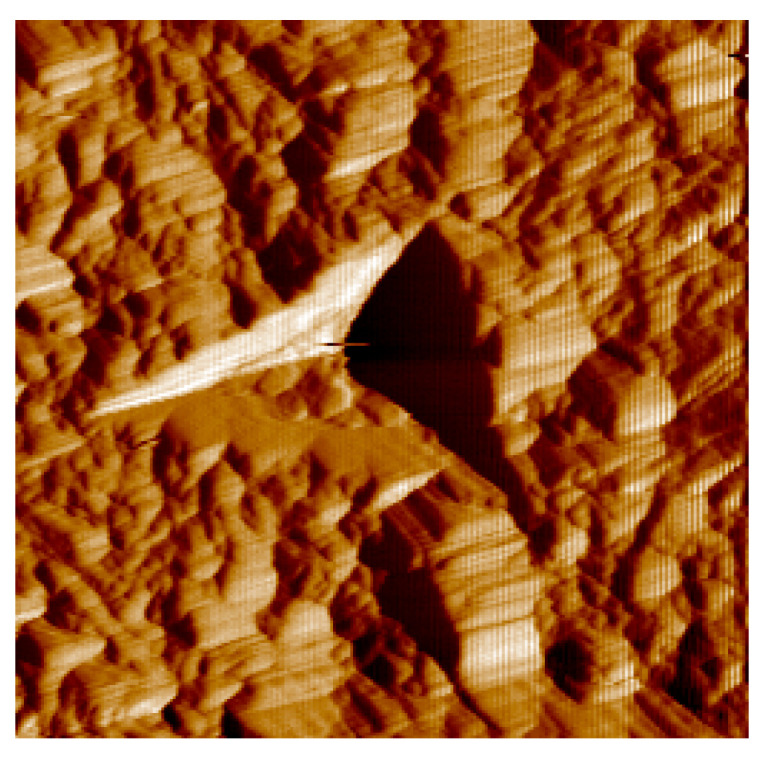
Nanoindentation microscopic image of fully annealed copper materials.

In this study, we measured five sets of fully annealed copper materials at different positions and then conducted inversion to solve material parameters, with the aim of checking to what degree the solution conformed to the physical constraints. The results in Table 4 show that the numerical solution is reasonable. (The five groups of nanoindentation tests in this study were conducted using a Hysitron TI 750 nanoindenter, with a constant maximum load mode (5 mN). The load was held for 10 s once the maximum load was attained, followed by the unloading phase) (see Figure 11).

The material parameters derived from inversion (hardening exponent *n* and yield strength *σ_y_*) were systematically compared with the data reported in the published literature for the same material (TSV-Cu) [19,34,35], and the results are presented in Table 5.

For most samples in this study (Samples 1, 2, 4, and 5), the inverted yield strength *σ_y_* spans from 35.70 to 46.14 MPa. This range is in excellent agreement with the value of 42.83 MPa reported by Li et al. [19] at the same annealing temperature (300 °C). It is also reasonably consistent with the strength range (47.91–51.83 MPa) of as-deposited electroplated copper cited in other studies [34,35]. This strong consistency provides robust external validation of the high accuracy and reliability of the machine learning inversion algorithm proposed in this study. Our results successfully validate the well-established principle that electroplated copper possesses a higher yield strength than fully annealed bulk copper, stemming from its fine-grained microstructure.

The hardening exponent *n* of the samples in this study (ranging from 0.5467 to 0.5665) is generally slightly higher than the range reported in the literature (0.49–0.52). This discrepancy may be attributed to the unique microstructures formed by the specific electroplating process employed (e.g., finer grain size), which results in enhanced work hardening capacity. It is worth emphasizing that the inversion method adopted in this study has undergone a sensitivity analysis and normalization range optimization (Section 3.1), leading to a significantly improved capability in identifying parameters such as the hardening exponent ***n*** and yield strength *σ_y_*. Thus, it can capture the mechanical responses that may arise from subtle differences in microstructure.

**Figure 12:**
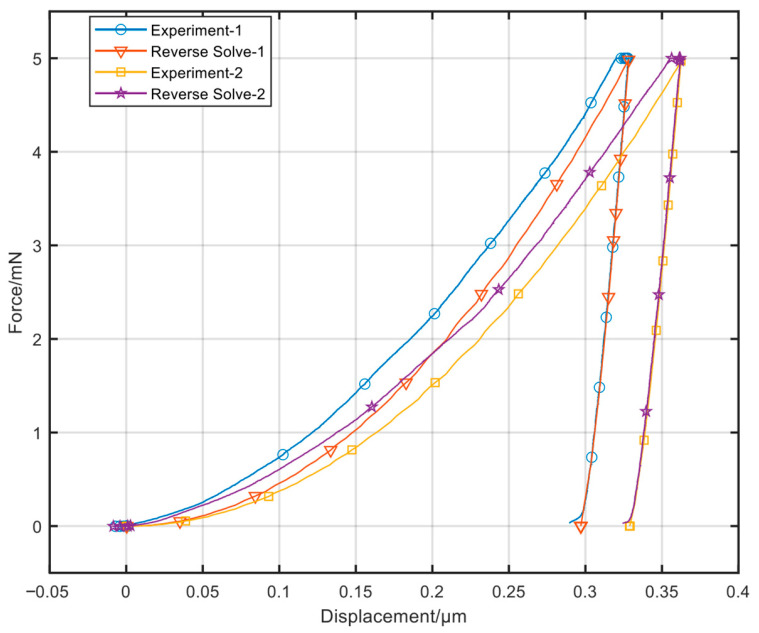
Comparison of *P*–*h* curves obtained through simulation based on material parameter values obtained through inversion and *P*–*h* curves obtained through experiments.

The yield strength *σ_y_* of Sample 3 (86.45 MPa) is notably higher. This is not an experimental or inversion error: the inverted results have passed physical rationality verification (Table 4), and the *P*–*h* curve reconstructed using these parameters shows a high degree of agreement with the experimental curve (Figure 12, with a convergence error of <0.1%). This finding precisely demonstrates that the proposed method possesses exceptional detection sensitivity and robustness in resolving the microstructural inhomogeneities of materials (e.g., regions with abnormally fine grains or higher impurity concentrations).

Through a systematic comparison with the literature data, we not only confirmed the reliability of the core results of this study but also highlighted the superiority of the proposed inversion method in resolving subtle differences in material mechanical properties. Our work supplements the existing database and provides a powerful tool for accurately characterizing the process–property relationship.

The trained model was used to perform inversion on the elastoplastic parameters of the selected fully annealed pure copper material. The measured values (Exp), reverse solution (RS) values, and simulation values obtained based on inversion parameters (virtual experimental value FEA) of the nanoindentation *P*–*h* curve were compared. The results are shown in Figure 12 and Table 6. The indentation *P*–*h* curve obtained in the test almost coincides with the curve plotted based on the parameter values obtained through inversion (or FFA), and the characteristic parameter error of the indentation curve is less than 0.1%.

### 3.3. Computational Efficiency

A comparison of the NN-LM method and traditional finite element iterative optimization is presented in Table 7.

The traditional FEM relies on repeatedly invoking finite element software for forward calculations, with a single forward simulation taking approximately 10 min. For parameter inversion, dozens to hundreds of iterations are typically required to achieve convergence, resulting in a total inversion time of hours or even longer.

The NN-LM method proposed in this study replaces finite element forward simulation with a well-trained neural network, reducing the forward calculation time from the minute to millisecond scale. Although the Levenberg–Marquardt (LM) algorithm requires an average of 200 iterations to converge to the target error (relative error < 1 × 10^−5^), the high efficiency of neural network forward propagation enables the entire inversion process for a single sample to be completed within 10 s, representing a speedup of more than two orders of magnitude compared with the traditional method.

### 3.4. Comparison of 3D Packaging Stress

**Figure 13:**
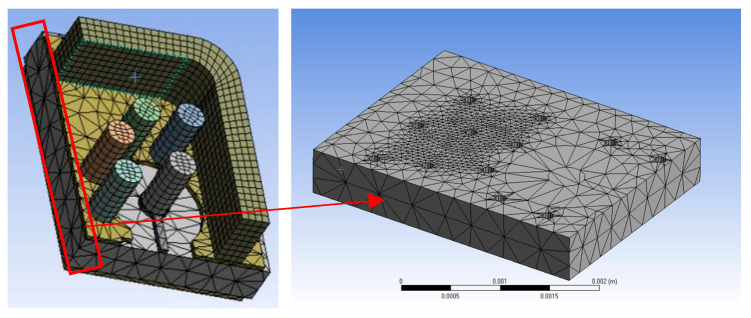
Geometric model of a 3D package with finite element mesh partitioning.

We used a finite element model of local weak positions in a high-density 3D SiP package formed using TCV to analyze and compare the differences between the method proposed in this paper and the simplified constitutive model based on engineering experience parameters regarding the influence on the stress of 3D packaging structures. The results are shown in Figure 13. The thermal expansion coefficient of the ceramic substrate was 7.1 ppm, the elastic modulus was 310 GPa, and Poisson’s ratio was 0.25. The grid was automatically partitioned into 28,570 elements and 53,843 nodes. WorkBench2023 was used to compare the stress conditions of copper-plated materials under the conditions of no structural constraints and constant temperature load (300 °C) using multilinear, bilinear, and power law constitutive models.

**Figure 14:**
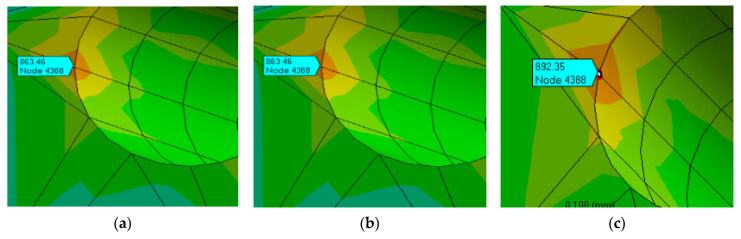

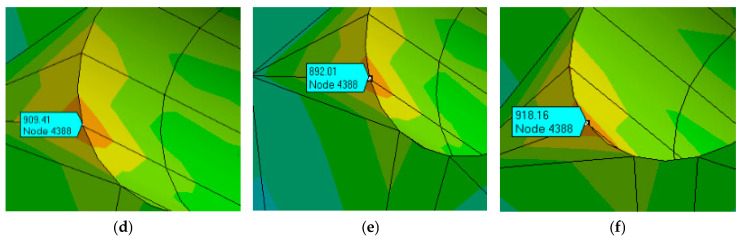
Stress distribution at copper-plated holes obtained from the simulation model. (**a**) The maximum principal stress at the node is 811.51 Mpa, (**b**) The maximum principal stress at the node is 863.59 Mpa, (**c**) The maximum principal stress at the node is 892.35 Mpa, (**d**) The maximum principal stress at the node is 909.41 Mpa, (**e**) The maximum principal stress at the node is 892.01 Mpa, (**f**) The maximum principal stress at the node is 918.16 Mpa.

The stress distributions of the materials obtained through model simulation are shown in Figure 14. The values of maximum principal stress at the same copper-plated hole obtained using different finite element models are extracted for comparison (Table 8). For the same grid node position, the stress obtained using the bilinear model is the highest, followed by that of the multilinear model, and the stress obtained using the power law model is the lowest (about 88%).

## 4. Conclusions

A neural network model for high-precision characterization of material power law constitutive model parameters was constructed based on characterization processing of the *P*–*h* curves plotted based on the data obtained in in situ near non-destructive testing of the parameters of microelectronic packaging materials using the nanoindentation testing technique. Then, a novel inversion algorithm was designed based on the neural network model for fast, accurate, and stable extraction of material mechanics parameters. The transformation process between the neural network model and overdetermined equation system inversion solution was worked out, and an inversion objective function with weighted factors and an improved LM algorithm with mask vectors were constructed to solve unknown inputs when some inputs and outputs are partially known. In addition, the least squares iteration of complex nonlinear equations between multiple variables was analyzed, along with the solution stability and uniqueness of inversion. By analyzing the differences between the influence of inputs on outputs regarding sensitivity, the normalization range of the modeling data was optimized to improve the stability of the reverse solution. The physical rationality of the reverse numerical solution was verified based on the proposed prior physical constraints of the elastoplastic constitutive curve’s intersection point. A comparative discussion of the material parameters derived from inversion against the data reported in the published literature not only verifies the reliability of the proposed method and the rationality of its results but also situates this study within the broader context of material characterization research, highlighting its significant value in the accurate elucidation of process–property relationships. A comparison with the results obtained through finite element simulation indicated that the proposed method can achieve an accuracy of <3% (95% confidence interval) in measuring material parameters, with a maximum error of less than 8%. A simulation of the stress of an actual packaging structure verified that the measured material parameters and the differences between material models have a significant impact on the stress, which provides useful guidance for optimization of the packaging process.

## Figures and Tables

**Table 1:** Parameter ranges of nanoindentation simulation.

Simulation Parameter	Unit	Minimum Value	Maximum Value	Number of Factor Levels
Elastic modulus (*E*)	GPa	60	160	6
Yield strength (*σ_y_*)	MPa	40	260	12
Strain strengthening index (*n*)	/	0	0.6	7
Displacement (*h*)	μm	0.3	0.6	4

**Table 2:** Adjustment of normalized hyperparameters.

Input Parameter	Original Value Range	Uniform Normalization Range	Independent Normalization Range
*E_r_* (Pa)	6 × 10^10^~16 × 10^10^	−1~1	−0.5~0.5
*n*	0~0.6	−1~1	−0.5~0.5
*σ_y_* (Pa)	40 × 10^6^~260 × 10^6^	−1~1	−0.2~0.2
*h_m_* (μm)	−0.6~−0.3	−1~1	−1~1
*C* (mN/μm^2^)	4.3~132	−1~1	−1~1
*S* (mN/μm)	92.9~847.4	−1~1	−0.2~0.2

**Table 3:** Comparison of sensitivity values before and after normalization range adjustment.

		Output Parameters of Unbalanced Sensitivity (Same Normalization Range)				Output Parameters of Balanced Sensitivity (Different Normalization Ranges)			
		*P_m_* (mN)	*W_u_* (mN·μm)	*W_l_* (mN·μm)	*h_f_* (μm)	*P_m_* (mN)	*W_u_* (mN·μm)	*W_l_* (mN·μm)	*h_f_* (μm)
Input Parameters	*S* (mN/μm)	0.019	0.0286	0.0096	0.0035	0.1474	0.0459	0.0195	0.0149
	*C* (mN/μm^2^)	0.2535	0.3037	0.1785	0.0947	0.3583	0.1728	0.0728	0.0802
	*h_m_* (μm)	0.1158	0.1854	0.3312	0.6012	0.096	0.0719	0.1409	0.2865
	*σ_y_* (Pa)	0.0232	0.0264	0.088	0.0248	0.1475	0.0285	0.1816	0.0587
	*n*	0.0495	0.1024	0.1251	0.0725	0.1105	0.0586	0.1351	0.0808
	*E_r_* (Pa)	0.0271	0.0982	0.0814	0.0653	0.0569	0.0851	0.0729	0.0849

**Table 4:** Comparison of physical rationality of numerical solutions.

Parameter	*n*	*σ_y_* (MPa)	*σ^R^_r_* (MPa, *n* = 0 Inversion)	*σ^E^_r_* (MPa, Calculated Using the Stress–Strain Intersection Formula)	Percentage Error (*σ^R^_r_* − *σ^E^_r_*)/*σ^E^_r_*
Sample 1	0.5529	46.14	47.75	51.29	6.9%
Sample 2	0.5665	40.78	48.57	52.32	7.2%
Sample 3	0.4830	86.45	48.48	53.24	8.9%
Sample 4	0.5467	39.06	42.37	42.10	−0.6%
Sample 5	0.5492	35.70	43.95	43.71	−0.6%

**Table 5:** Comparison of inverted and literature data for yield strength (*σ_y_*) and hardening exponent (*n*) of electroplated copper.

Data Source	Hardening Exponent (*n*)	Yield Strength *σ_y_* (MPa)
[19]	0.5212	42.83
[34]	0.4892	47.91
[35]	0.5093	51.83
This Study (Samples 1–5)	0.4830~0.5665 (Mean: 0.5397)	35.70~86.45 (Sample 3: 86.45; Mean of Others: 40.42)

**Table 6:** Comparison of accuracy in finite element simulation with input numerical solutions.

Parameter		Characteristic Parameters of *P*–*h* Indentation Curve							Material Parameters Obtained Through Inversion	
		*f_m_* (μm)	*C* (mN/um^2^)	*S* (mN/μm)	*P_m_* (mN)	*W_l_* (mN·μm)	*W_u_* (mN·μm)	*h_f_* (μm)	*n*	*σ_y_* (MPa)
Sample 1	Exp.	0.3281	46.44	202.4	4.999	0.4793	0.0675	0.2971	—	—
	RS	0.3281	46.44	202.4	4.995	0.4787	0.0671	0.2971	0.5529	46.14
	FEA	0.3283	46.67	200.4	4.981	0.4747	0.0673	0.2970	0.5529	46.14
	relEr.	−0.06%	−0.49%	1.00%	0.28%	0.84%	−0.30%	0.03%		
Sample 2	Exp.	0.3619	38.17	176.6	4.999	0.5303	0.0728	0.3287	—	—
	RS	0.3619	38.17	176.6	4.981	0.5290	0.0718	0.3287	0.5492	35.70
	FEA	0.3624	38.18	178.5	4.971	0.5255	0.0722	0.3290	0.5492	35.70
	relEr.	−0.14%	−0.03%	−1.06%	0.20%	0.67%	−0.55%	−0.09%		

**Table 7:** Comparison of the NN-LM method and traditional finite element iterative optimization.

Method Type	Time Per Forward Calculation	Number of Iterations Per Inversion	Total Inversion Time (Single Sample)	Test Environment
Traditional FEM Iterative Optimization [11]	~10 min	Dozens to hundreds	Hours to tens of hours	Single-core 2.4 GHz
NN-LM Method (This Study)	Millisecond scale (forward prediction)	Average 200	<10 s	Single-core 2.4 GHz

**Table 8:** Comparison of accuracy between three models in the finite element simulation with input numerical solutions.

Group Number	Cu Material Model		Maximum Principal Stress of Node (MPa)	Normalized Value
(1)	Power law model PowerLaw (*n* = 0.545, *σ_y_* = 5.83 × 10^7^)		811.51	0.88
(2)	Multilinear model	pad Cu Multilinear	863.59	0.94
(3)		pad Cu MultilinearSC-7	892.35	0.97
(4)		pad Cu MultilinearSC-7-2	909.41	0.99
(5)		pad Cu WEIKAO-11	892.01	0.97
(6)	Bilinear model pad Cu Bilinear (normalized reference)		918.16	1.00

## Data Availability

The data presented in this study are available from the corresponding author upon request.
